# The Role of Mobile Genetic Elements in Virulence Factor Carriage from Symptomatic and Asymptomatic Cases of Escherichia coli Bacteriuria

**DOI:** 10.1128/spectrum.04710-22

**Published:** 2023-05-17

**Authors:** Grace Morales, Benjamin Abelson, Seth Reasoner, Jordan Miller, Ashlee M. Earl, Maria Hadjifrangiskou, Jonathan Schmitz

**Affiliations:** a Department of Pathology, Microbiology, and Immunology, Vanderbilt University, Nashville, Tennessee, USA; b Department of Urology, Vanderbilt University Medical Center, Nashville, Tennessee, USA; c Vanderbilt Institute for Infection, Immunology, and Inflammation, Vanderbilt University, Nashville, Tennessee, USA; d Infectious Disease and Microbiome Program, Broad Institute, Cambridge, Massachusetts, USA; University of Pittsburgh School of Medicine

**Keywords:** AMR, antibiotic resistance, MGE, mobile genetic elements, UPEC, urinary tract infection, virulence factors

## Abstract

Uropathogenic Escherichia coli (UPEC) is extremely diverse genotypically and phenotypically. Individual strains can variably carry diverse virulence factors, making it challenging to define a molecular signature for this pathotype. For many bacterial pathogens, mobile genetic elements (MGEs) constitute a major mechanism of virulence factor acquisition. For urinary E. coli, the total distribution of MGEs and their role in the acquisition of virulence factors is not well defined, including in the context of symptomatic infection versus asymptomatic bacteriuria (ASB). In this work, we characterized 151 isolates of E. coli, derived from patients with either urinary tract infection (UTI) or ASB. For both sets of E. coli, we catalogued the presence of plasmids, prophage, and transposons. We analyzed MGE sequences for the presence of virulence factors and antimicrobial resistance genes. These MGEs were associated with only ~4% of total virulence associated genes, while plasmids contributed to ~15% of antimicrobial resistance genes under consideration. Our analyses suggests that, across strains of E. coli, MGEs are not a prominent driver of urinary tract pathogenesis and symptomatic infection.

**IMPORTANCE** Escherichia coli is the most common etiological agent of urinary tract infections (UTIs), with UTI-associated strains designated “uropathogenic” E. coli or UPEC. Across urinary strains of E. coli, the global landscape of MGEs and its relationship to virulence factor carriage and clinical symptomatology require greater clarity. Here, we demonstrate that many of the putative virulence factors of UPEC are not associated with acquisition due to MGEs. The current work enhances our understanding of the strain-to-strain variability and pathogenic potential of urine-associated E. coli and points toward more subtle genomic differences distinguishing ASB from UTI isolates.

## INTRODUCTION

An estimated 50% of women will experience at least one urinary tract infection (UTI) in their lifetime, making UTIs one of the most prevalent human bacterial infections (1). Recurrent UTIs are likewise common and, in many cases, are caused by the same isolate that caused the original infection (2, 3). Overall, Escherichia coli accounts for >75% of UTIs (1, 4). A universal commensal of the gut, E. coli can colonize the urogenital environment and ascend the bladder to elicit disease (5), including residing in quiescent bladder reservoirs between episodes (1, 4, 6). These “uropathogenic” E. coli (UPEC) strains typically cluster in phylogroups B2 and D and lack the distinguishing molecular characteristics of diarrheagenic pathotypes such as enterohemorrhagic or enterotoxigenic E. coli (EHEC and ETEC, respectively) (5, 7, 8).

Another factor complicating our ability to define UPEC molecularly is the common phenomenon of E. coli asymptomatic bacteriuria (ASB) (9–11). In ASB, a clean-catch urine sample with a high bacterial burden (typically ≥10^5^ CFU/mL) is observed, but without the patient experiencing UTI-associated signs or symptoms. Strains of E. coli isolated from cases of ASB have not been distinguished from UTI-associated isolates in terms of specific defining virulence factors, and they cluster genetically in the same B2 and D clades (9–15). In general, B2 and D strains are known to harbor more virulence factors than other gut-commensal phylogroups, such as A, B1, and C (16, 17). In addition to classifying strains by clade, E. coli strains can be differentiated through their multilocus sequence type (MLST). Many UPEC and ASB isolates cluster in sequence type 73 (ST73), ST69, and ST131 (18, 19). Specific MLSTs appear to carry larger quantities of antimicrobial resistance genes, such as the multidrug-resistant sequence type 131 (20–23). Nevertheless, across both clade- and MLST-based schema, urine-associated E. coli strains are diverse genotypically and phenotypically, with no specific virulence factor signature that clearly defines UPEC (10, 12, 24–27). The current lack of organismal criteria for ASB- and UTI-associated strains can lead to a clinical dilemma when a patient’s symptomatology is ambiguous or of unclear etiology, including in the overprescription of antibiotics. For most clinical scenarios, current guidelines do not indicate antibiotic therapy for cases of ASB (28).

For diverse species, the bioinformatic identification of mobile genetic elements (MGEs) has enhanced our knowledge of how these elements shape bacterial physiology and pathogenicity (29–33). MGEs represent an important source of virulence factors, antimicrobial resistance genes, and general genetic diversity (34–41). For diarrheagenic E. coli, MGEs can represent a pathotype-defining mechanism of virulence factor acquisition, such as the case of Shiga toxin in EHEC (42–44). While carriage of these enteric virulence factors is uncommon in urinary E. coli (45), other virulence-associated genes may be concentrated within pathogenicity islands (PAI), large genomic tracts that can be mobilized via conjugation (46, 47). Several PAI have been identified in UPEC strains, although they still do not constitute a defining feature for the pathotype (48–51).

To these ends, the current work systematically characterizes the presence of additional MGEs in urinary E. coli—including from cases of both asymptomatic bacteriuria and symptomatic UTI—along with the association of these MGEs with virulence factors and AMR-associated genes. As no discrete genes currently define strains of E. coli as UPEC (10, 52), we sought to determine if MGE carriage (both in amount and type) might help characterize this pathotype via a differential association with symptomatic (i.e., UTI) and ASB cohorts (44, 53, 54). Overall, we wished to determine if (i) strains from UTI cases harbored more MGEs than ASB-associated strains and (ii) if the MGEs within UTI-associated strains encode a greater number of total virulence factors and AMR genes than MGEs from ASB-associated strains. Although we did not anticipate any singular MGE-encoded genes to define these cohorts, this work may clarify if the global MGE landscape of urinary E. coli correlates with patient symptomatology.

Specifically, we applied Illumina whole-genome sequencing and bioinformatic analyses to a diverse collection of E. coli from patients with UTI and ASB. We sought to characterize the relationship between the clinical presentation of these individuals (UTI versus ASB) and targeted genetic features of their associated strains. Specifically, we compared their phylogenetic distribution, the carriage of MGEs (prophage, plasmids, and transposons), and the corresponding location of virulence factors associated with uropathogenesis, along with antimicrobial resistance genes. Among our findings, we observed that the per-strain abundance of virulence factors showed phylogroup-specific trends (independent of whether the strain derived from UTI versus ASB) and that plasmids constituted a prominent source of antimicrobial resistance determinants. These groups of MGEs constituted only a very modest source of virulence factors for both UTI- and ASB-associated strains. Notably, we observed no correlation of MGEs or virulence factors with the presence of symptoms in the source patient. These findings support the notion that the definition of the UPEC pathotype, including what might distinguish strains that elicit symptomatic infection and asymptomatic colonization, lies outside the individual or cumulative presence of MGEs and virulence factors.

## RESULTS

### Clinical and phylogenomic characterization of urinary E. coli.

**FIG 1:**
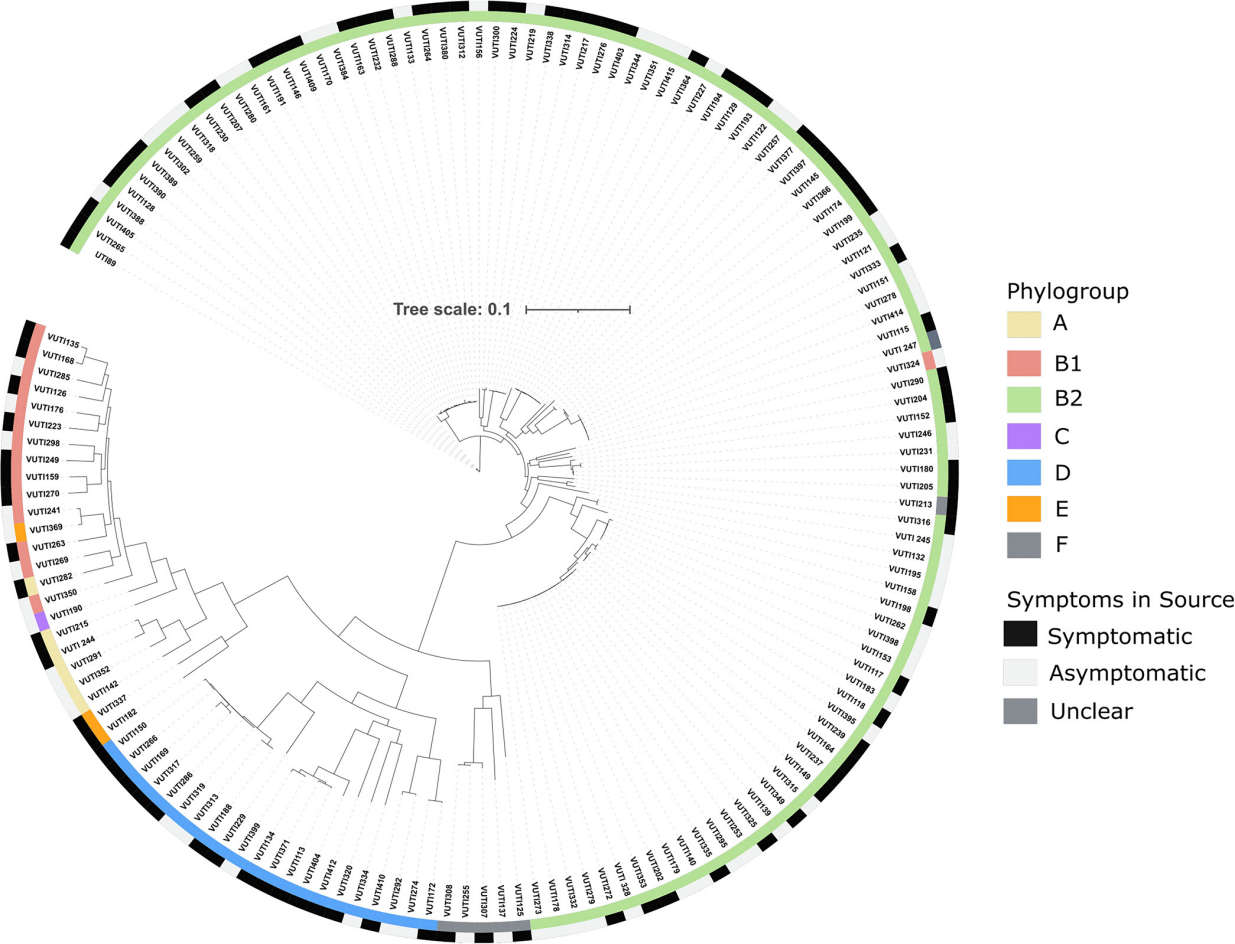
Phylogenomic analysis of 151 E. coli isolates from urine. A core genome alignment was completed using Parsnp. A maximum likelihood tree was constructed from the alignment and was visualized using iTOL. The outer ring of the phylogenomic tree indicates the presence of symptoms in the source patient. The phylogroup or clade of the strain is indicated in the inner ring, as identified through ClermontTyping.

To evaluate if MGE carriage is a distinguishing feature of UPEC, we conducted whole-genome sequencing of 151 urine-isolated E. coli strains that were previously characterized by their biofilm and metabolic traits (see Table S1 in the supplemental material), as well as the clinical scenarios of their source patients (52, 55). These 151 strains originated from individuals with diverse clinical presentations, including ASB (63/151, 41.7%), cystitis (77/151, 50.9%), pyelonephritis (6/151, 3.9%), bacteremia (3/151, 1.9%), and one unclear case (1/151, 0.66%) (Fig. 1, Table S1). For this study, we binned all the symptom-associated strains (cystitis, pyelonephritis, bacteremia, unclear) together and compared the genetic features of these strains to those of the ASB group.

For each strain, DNA was extracted and subjected to Illumina short-read sequencing, and reads were used in *de novo* assembly using SPAdes; assembly statistics are highlighted in Table 1 and Table S1 (56). From the sequencing data, we applied ClermonTyping to assign clades (57). A phylogenetic tree was built based on the core genome using Parsnp (58) and visualized using iTOL (59).

**TABLE 1:** Summary of assembly statistics

Trait	Value
Mean GC (%)	50.59
Mean no. of contigs	92.65
Mean *N*_50_	20,9951.63
Mean completeness (%)	99.43
Mean largest contig (bp)	520,685.87
Mean contamination (%)	0.672

The isolates were representative of phylogroups A, B1, B2, C, D, E, and F, with 120/151 (79.5%), belonging to B2 and D (Fig. 1, Table S1), an observation consistent with previous work (12–14). Multilocus sequence types (MLSTs) varied among the sequenced strains, with only 4/151 strains harboring an MLST not found in the PubMLST database (60) (Fig. S1). Consistent with reports indicating ST131 as the most prevalent MLST in recent years (61), the MLSTs with the highest frequency in our data set, 27/151 isolates (17.8%), are of the ST131 MLST, (Fig. S1, Table S1). Of the ST131 isolates, 11/27 (40.7%) came from patients with a UTI. Overall, our analysis demonstrated that ASB- and symptom-associated strains were similarly distributed among phylogroups and MLSTs (Fig. 1).

### Prevalence of virulence factors across urinary E. coli.

To characterize the presence of virulence factors within the 151 clinical isolates, we curated a list of 112 virulence-associated genes that represent 52 Escherichia coli virulence factors frequently found in uropathogenic and diarrheagenic pathotypes. Since we found that the commonly used VirulenceFinder database was not well tuned for UPEC, we supplemented it with known UPEC virulence factors based on the literature and removed virulence factors not generally found in UPEC isolates (Tables S2 and S3 and Supplementary Files) (25, 40, 51). Several virulence factors associated with diarrheagenic disease were kept as controls, as we did not expect to find these genes in high prevalence in our cohort. We considered all genes (here termed virulence-associated genes) that are required to produce a functional virulence factor (here referring to a fully functional protein or protein complex for a virulence factor). Of note, these include genes that code adhesive organelles, such as type 1 pili (*fim*), as well as iron acquisition systems, toxins, adhesins, and immune modulating proteins (Tables S2 and 3 and Files S1 to S4).

**FIG 2:**
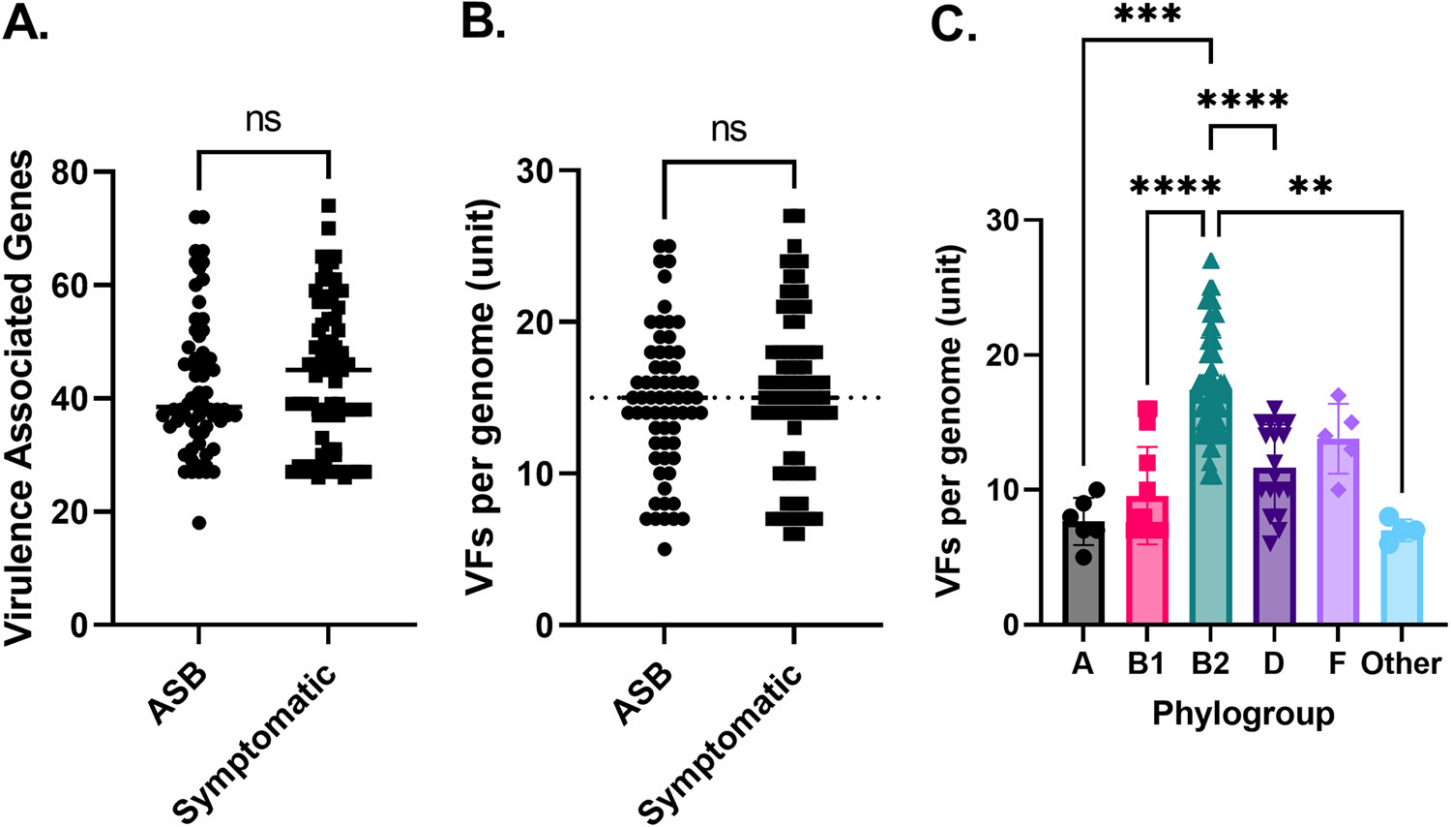
Identification of virulence-associated genes and complete virulence factors in urine-isolated E. coli. (A) Virulence-associated genes were identified using BLAST and counted individually (*P* > 0.05 by Mann-Whitney test). (B) Virulence factor units were considered present if all virulence-associated genes for a given factor were identified (*P* > 0.05 by Mann-Whitney test). (C) Virulence factors were identified more frequently in the B2 phylogroup than in the A, B1, D, and Other (encompassing C and E) groups (considered significant if *P* < 0.05; Kruskal-Wallis test with Dunn’s multiple comparisons).

We utilized BLAST (62) to identify the presence of these genes within the genome assemblies. This approach identified a total of 6,591 genes encoding virulence factors (Fig. 2A and B). These genes account for 2,284 predicted functional virulence factors. When considering how many functional virulence factors were carried by each strain, we found an average of 15 virulence factors per isolate, with a range among all isolates of 5 to 25. When categorized by UTI versus ASB, the UTI isolates had an average of 15 predicted functional virulence factors with a range of 6 to 25, while ASB had an average of 14 virulence factors per strain with a range of 5 to 25. There was no statistical difference in number of virulence factors in strains from ASB versus symptomatic patients when tested with Mann-Whitney *t* tests. We compared the number of virulence factors among phylogroups and found that clade B2 carried more virulence factors (per strain, *P* < 0.05 with Kruskal-Wallis test) than all other clades except F (Fig. 2C).

In addition to the total number of virulence factors per strain, we also inquired whether the presence of any particular factor was associated with the clinical presentation of the patient, which we tested via Chi-squared test (Fig. S3). Indeed, the number of individual virulence-associated genes or complete virulence factors could not be significantly associated with UTI- versus ASB-associated isolates (Fig. S3 and Tables S2, S3, and S5).

### Prevalence of antimicrobial resistance genes across urinary E. coli isolates.

**FIG 3:**
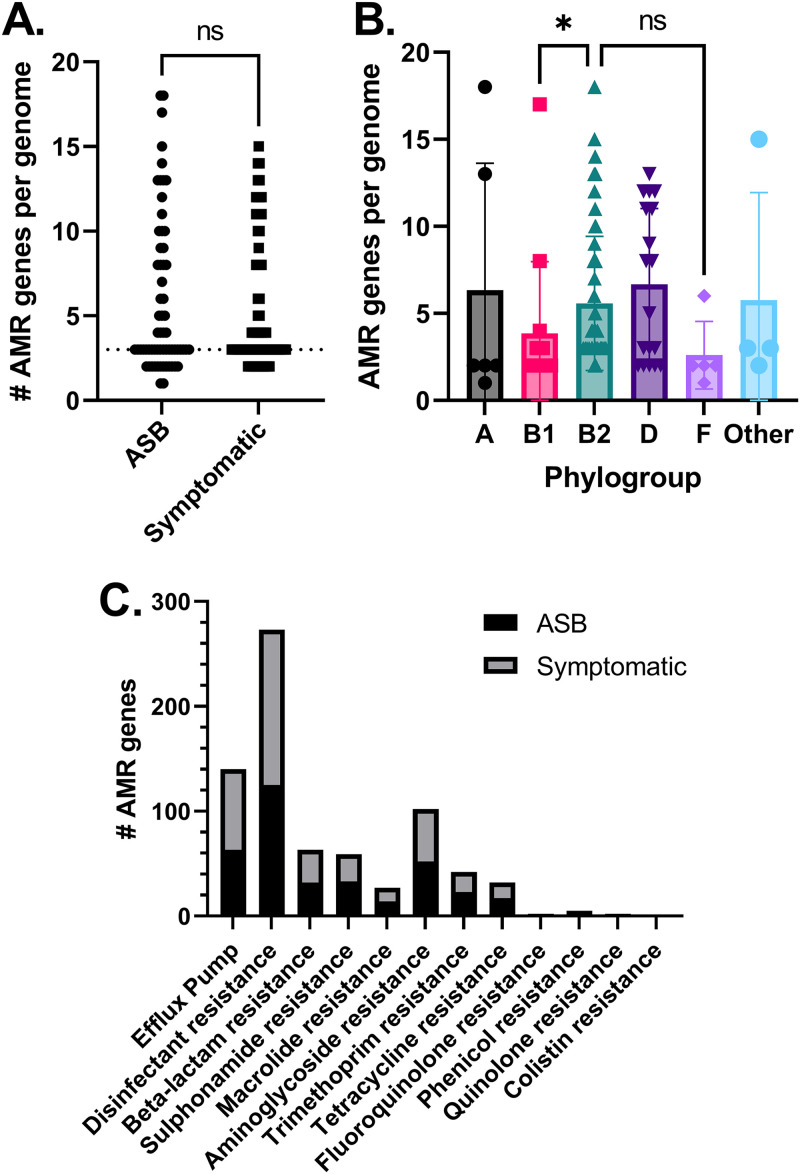
Antimicrobial resistance (AMR) genes identified in urine-isolated E. coli isolates. (A) AMR genes were equally distributed among symptomatic and asymptomatic patients (*P* > 0.05 by Mann-Whitney test). (B) AMR genes were slightly more significantly associated with the B2 phylogroup than the B1 and F groups (*P* < 0.05 with Kruskal-Wallis test with Dunn’s multiple comparisons). (C) Symptomatic patients additionally did not carry more resistance genes associated with any drug class (*P* > 0.05 by 2-way ANOVA).

Given the prominence of antimicrobial resistance in uropathogens, with up to 90% of isolates being resistant to at least one agent, we explored the distribution of antibiotic resistance genes (63–65). We first employed ResFinder (40) to identify genes associated with antimicrobial resistance (AMR) in our cohort (Table S8). The ResFinder database includes enzymes, efflux pumps, and alternative alleles (Tables S4, S6, and S8). In total, we identified 783 instances of AMR genes across 48 distinct AMR alleles across all isolates. Each strain in the cohort carried at least one AMR gene, with a median of 3 and a cohort range of 1 to 18 (Table S4). UTI-associated isolates had a median of 3 AMR genes per strain, with a range of 2 to 14. The ASB strains also had a median of 3 AMR genes per strain, with a range of 1 to 18 (Fig. 3A). The most commonly identified genes were *mdfA* (multidrug efflux) and *sitABCD* (peroxide resistance). The most prevalent predicted resistance was to the sulfonamide, β-lactam, aminoglycoside, and trimethoprim drug classes (Table 2). Phylogroup B2 had significantly more AMR genes than phylogroups B1 and F (Fig. 3B). Similar to other reports, we observed that ST131 carried significantly more AMR genes than most other sequence types in the data set (Fig. S2) (23, 61, 66–68).

**TABLE 2:** Most frequently predicted drug class resistance using ResFinder

Drug class example	No. of isolates (%)
Aminoglycoside resistance	103 (68.2)
β-Lactam resistance	64 (42.3)
Sulfonamide resistance	60 (39.7)
Trimethoprim resistance	43 (28.4)
Tetracycline resistance	33 (21.8)
Macrolide resistance	29 (19.2)

Neither the burden of AMR genes (Fig. 3A and Fig. S3) nor predicted resistance to specific drug classes (Fig. 3C) correlated with the presence of symptoms in the source patient when tested with Mann-Whitney *t* test and two-way analysis of variance (ANOVA) test, respectively (Table S3).

### Prophages are not a significant source of virulence factors or antimicrobial resistance in urinary E. coli.

As prophages can be instrumental in the virulence of bacterial pathogens (36, 69, 70), we next queried prophage sequences in our cohort of 151 strains to investigate whether prophage features might likewise correlate with the clinical symptomology of urinary E. coli. PHASTER was employed to predict prophage elements within the assemblies (71). This tool assigns a score to the elements identified to predict completeness of the prophage element based on the length of the prophage sequence, the presence of structural proteins, and the presence of an attachment site. Of the 946 prophage elements identified by PHASTER, only 403 (42.6%) were predicted to be complete sequences (Fig. S3). Among these, the enterobacterial phage mEp460 (NC_019716) was the most prevalent, present in 29/151 isolates (Table 3).

**TABLE 3:** Top five most frequently identified complete prophage elements

Closest phage (GenBank accession no.)	No. of instances
Enterobacterial phage mEp460 (NC_019716)	29
Enterobacterial phage P88 (NC_026014)	22
Enterobacterial phage BP-4795 (NC_004813)	15
Shigella phage SfII (NC_021857)	13
*Pectobacterium* phage ZF40 (NC_019522)	12

**FIG 4:**
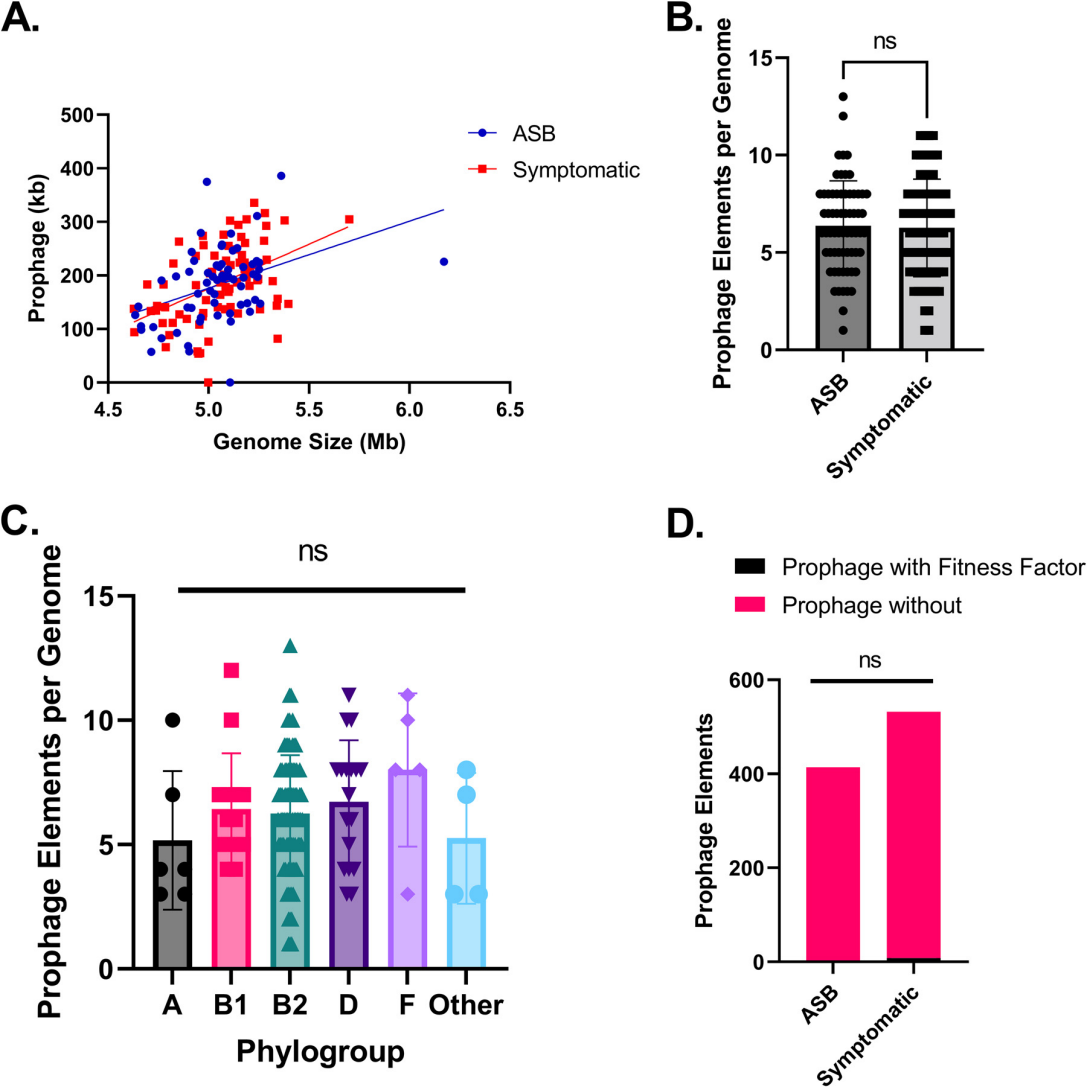
Prophages are not a significant source of virulence factors or AMR genes in urine-isolated E. coli. Prophage elements were identified using PHASTER. (A) The amount of prophage, in kilobases, is positively correlated with the genome size of the strain (linear regression analysis). (B and C) Prophage elements, regardless of completeness, are not significantly associated with (B) the presence of symptoms in the source patient or (C) the phylogroup of the strain (*P* > 0.05 by Mann-Whitney test and Kruskal-Wallis test with Dunn’s multiple comparisons, respectively). (D) The majority of prophage elements do not carry virulence genes or AMR genes, and carriage of these genes by prophages is not significantly associated with the presence of symptoms (*P* > 0.05 by 2-way ANOVA).

A positive Pearson’s coefficient was observed between the genome size of an isolate and the total, in kilobases, prophages carried (Fig. 4A), indicating a correlation between genome size and total prophages carried. Regardless of completeness, the number of prophage elements within a given genome was between 0 and 13, with a median of 6 (Fig. 4B) (Mann-Whitney test). Like the descriptions above, this number was observed to be independent of the presence of symptoms in the source patient (Fig. 4B) or phylogroup of the strain (Fig. 4C) (*P* > 0.05 with Mann-Whitney and Kruskal-Wallis tests).

We next checked if the prophages’ locations overlapped the locations of virulence factors and AMR genes to gauge the carriage of these genes by the prophage elements. Only 43/6,591 virulence-associated genes identified were predicted to be carried by prophage elements in 10 strains, suggesting that prophages are not a major contributor to virulence factor acquisition in UPEC. These 43 virulence-associated genes that overlapped prophage elements encoded five fully functional virulence factors across five strains (one per strain), including the adherence appendages encoded by the *afa*, *dra*, and *fim* gene clusters (Table S5). Across all 151 strains, however, these particular virulence factors were more frequently associated with chromosomal DNA, rather than with a prophage element (Table S5). When we checked prophage elements with the antibiotic resistance factors, only two instances including genes *blaTEM-1A* and *formA* were associated with prophages across all strains (Table S6).

### Plasmids contribute to AMR and virulence factor acquisition.

**FIG 5:**
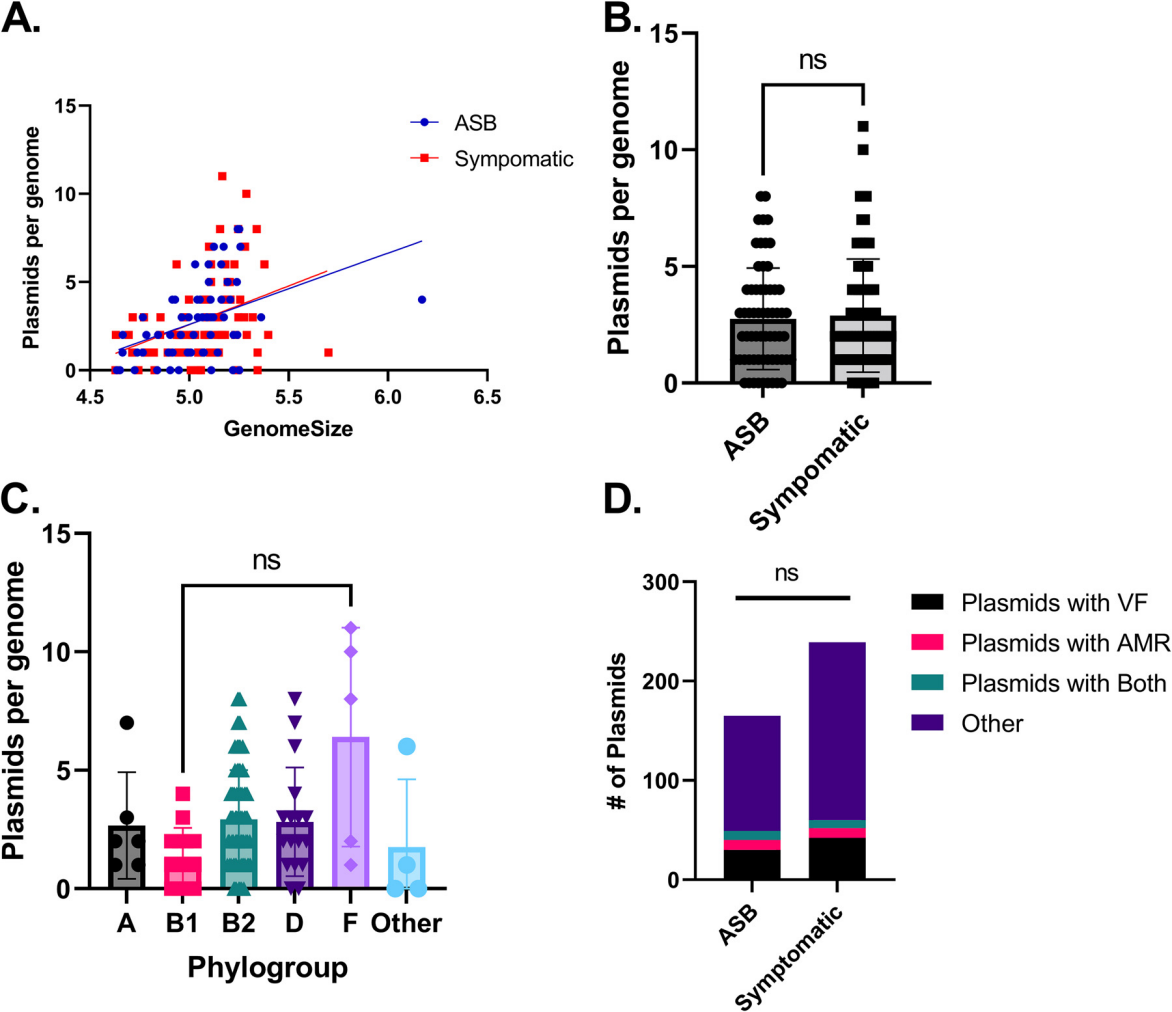
Plasmids contribute to virulence factor and AMR gene acquisition in urine-isolated E. coli. Plasmid sequences were identified using plasmidSPAdes. Virulence-associated genes and AMR genes were identified using BLAST and ResFinder, respectively. (A) Plasmids are positively correlated with the size of the genome of the strain (linear analysis). (B) Plasmids are not significantly associated with the clinical presentation of the source patient (*P* > 0.05 by Mann-Whitney test). (C) Plasmids are significantly increased in the F phylogroup compared to B1, but not significantly different in any other comparison (*P* < 0.05 Kruskal-Wallis Test with Dunn’s multiple comparisons). (D) Plasmid composition is similar between ASB and symptomatic strains (*P* > 0.05 by 2-way ANOVA; row factor).

We next evaluated the presence of plasmids and their correlation with AMR and virulence factors. Plasmid sequences were assembled from the read data using plasmidSPADES (56). We further preformed *in silico* replicon typing and identified the NCBI nearest neighbor using PlasmidFinder and MOB-suite (72, 73). We found incompatibility plasmids (Inc) and colicin-conferring plasmids (Col) to be the most abundant, with 92 and 86 identified plasmid sequences, respectively (Table 4). Between these two plasmid types, this accounts for 43.8% of all identified plasmid sequences in the cohort (Table S7). However, 61% of the plasmids had no known replicon type (Table 4). Interestingly, despite the prevalence of colicin-conferring plasmids, we detected only one instance of a colicin resistance gene in the data set (Fig. 3C). A median of 2 plasmids were predicted per strain, with a range of 0 to 11 predicted plasmids (Table 4). We found that the number of predicted plasmids identified was positively correlated with genome size (Fig. 5A). We identified AMR genes by using the extracted predicted plasmid sequences in ResFinder (40, 74). Virulence-associated genes were identified within these sequences using BLAST, using the sequences in Files S1 to S4. Within the plasmid sequences, we identified 112 instances of antibiotic resistance genes and 209 virulence-associated genes. This corresponded to 14.3% of the total AMR genes and 3.2% of virulence-associated genes (Tables S5 and S6). The total number of predicted plasmids was independent of the presence of symptoms in the source patient and independent of the phylogroup (Fig. 5B).

**TABLE 4:** Summary of plasmid assembly statistics

Trait	Value
Total plasmids identified	498
Median plasmids per strain (range)	2 (0–10)
Avg size (bp)	10,442
Most commonly identified plasmid type	Inc plasmids (92/406)
No. of unknown plasmid types	248/406 (61%)

We next investigated how plasmid-encoded virulence factors or AMR genes were distributed among UTI strains or ASB strains. Plasmids were not associated with the presence of symptoms, regardless of how many virulence factors or antimicrobial resistance genes they contained (Fig. 5D). This indicates that acquisition of these fitness factors through plasmids is not associated with symptomatic colonization. Additionally, we found that plasmids carrying AMR genes rarely carried virulence factors (Fig. 5D).

### Other mobile genetic elements contribute to virulence factor acquisition.

**FIG 6:**
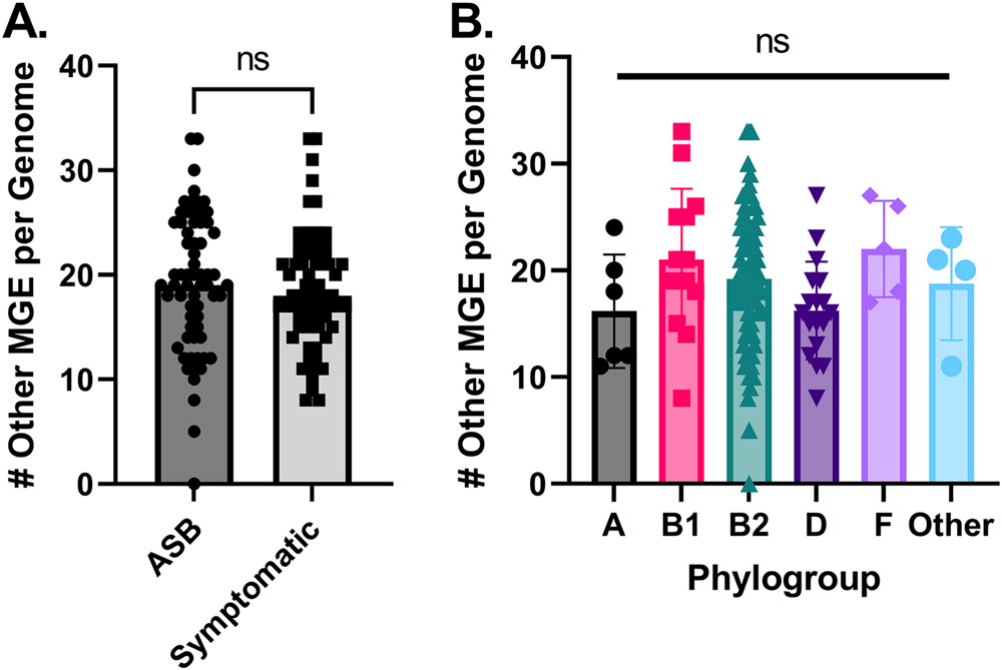
Other MGEs do not contribute to virulence factor (VF) or AMR genes in urine-isolated E. coli. Other MGEs were identified using MobileElementFinder, and the locations of these mobile elements were cross-correlated with the locations of virulence-associated genes and AMR genes previously identified. (A) Mobile elements (including insertion sequences, transposons, and integrative and conjugative elements) are not associated with symptomatic colonization (*P* > 0.05 by Mann-Whitney test). (B) Other MGEs are found independent of phylogroup (Kruskal-Wallis test with Dunn’s multiple comparisons).

We next searched for additional MGEs using MobileElementFinder, which identifies insertion sequences and integrative and conjugative elements (ICE), as well as transposons, from sequence data. We identified 2,898 MGEs within our strains, with the majority of these MGEs being insertion sequences (Fig. S5). Once again, these other MGEs were carried without a clear association with the clinical presentation and phylogroup (Fig. 6A and B).

The locations of these additional MGEs were likewise cross-correlated with the location of virulence factors and AMR genes, similar to the prophage analyses described above. We considered there to be an association if a gene was identified within 31 kb of the MGEs based on the largest known conjugative transposon in the family *Enterobacteriaceae* (29, 75). By considering this additional flanking sequence, we took into account the potential for linkage between a mobilized element and an adjacent chromosomal sequence. We identified no virulence-associated genes or AMR genes associated with other MGEs (Tables S5 and S6). We also considered the possibility that genes might be mobilized by an MGE that was not annotated due to being near the end of an assembled contig. Within our data set, we found that 59% of the AMR genes and 40% of virulence genes were annotated within 31 kb of the end of a contig. Thus, while we found no direct associations of AMR genes and these other MGEs, we cannot conclude that MGEs do not contribute to acquisition of these features in urinary E. coli. Nevertheless, in this context, our data suggest that MGEs do not constitute a defining feature to distinguish ASB from symptomatic strains.

## DISCUSSION

In this work, we investigated how MGEs shape fitness factor acquisition in Escherichia coli isolated from the urine of UTI and ASB patients, with the goal of determining whether MGEs could distinguish these two groups of clinically important isolates. While UPEC does not have a genetic signature associated with it yet, MGEs have been shown to be helpful in pathotype assignment in E. coli, such as in the case of the prophage-carried Shiga toxin (43, 76, 77). With UTI being one of the most prevalent bacterial infections in humans, it becomes imperative to understand what features distinguish uropathogenic E. coli from others within the same phylogroup.

Overall, we conclude that MGEs moderately contribute to antimicrobial resistance and that virulence factors seem to be clade dependent and not linked with antimicrobial resistance gene acquisition. We determined that approximately 14% of antimicrobial resistance genes were acquired through plasmids, which covered some of the most commonly plasmid-acquired antibiotic drug classes (Table S6) (78, 79). Several studies have indicated an increase in extended-spectrum β-lactamase-producing E. coli from patients with bacteriuria. We corroborate this finding and show a similar prevalence of ESBL genes as in other studies (80).

We report that the incidence of virulence factors mobilized by any element is less than 10%, suggesting that virulence factors are encoded in the chromosome and arise through pathogenicity islands as well as through clade effects. This is supported by the distribution of virulence factors by phylogroup (Fig. 3D). Phylogroups B2 and D statistically carry more virulence factors than the other phylogroups, which is corroborated by other studies (16, 17, 81). While phylogroups E and B1 are most frequently associated with highly virulent EHEC strains (15, 81), we identified both in our data set. Both of these traditionally highly virulent groups contained fewer virulence factors than the B2 and D phylogroups.

Unlike EHEC or STEC, mobile genetic elements do not define UPEC. Each mobile element and fitness factor was tested against the presence of symptoms in the source patient, none of which were found to correlate with UTI. Other studies have shown that in itself, integration and maintenance of prophage elements can impact carbon metabolism, predation, and respiration of the bacteria (76, 82, 83). Prophages might impact how UPEC interacts with the host bladder environment by influencing how it metabolizes and navigates the hypoxic bladder. Given the diverse nature of prophage elements identified in strains from both asymptomatic and symptomatic patients, integration sites and additional genes carried by prophages could play an important role in discerning how prophages might impact bladder colonization and subsequent infection (70, 84).

This is the first study to look at the correlation of MGEs and fitness factors in UPEC, although it could potentially still underestimate the MGEs’ identification due to the nature of the short-read sequencing, (85, 86). However, these predictions and analyses provide insights into how MGEs shape the virulence landscape of urine-isolated E. coli. Additionally, this work focuses on the genomics of the bacteria colonizing the bladder of the human host, agnostic of the effect the host’s immune system might have. It is known that bacteria that do not elicit as strong an innate response, typically through Toll-like receptor 4 (TLR4), will typically cause ASB, while strains eliciting a strong immune response will result in a symptomatic UTI (87–89). It is possible that other genes carried by these MGEs could impact how UPEC modulates the immune response through metabolism changes or regulation of virulence factor genes already in the genome.

In summary, this study provides a basis for how UPEC, including strains from asymptomatic bacteriuria, might acquire fitness factors. While we show that MGE carriage and virulence factors do not discretely define UPEC, this work suggests that a more nuanced molecular signature, such as in polymorphisms present in their core genome, might exist to identify urine-isolated strains with pathogenic potential.

## MATERIALS AND METHODS

### Strain information.

Escherichia coli strains used in this study were isolated from positive urine samples under Vanderbilt institutional review board (IRB) approval no. 151465 as previously described; these specimens/organisms were obtained under the auspices of the MicoVU clinical microbial biobanking program (52). A list of strains and relevant information (phylogroup, clinical scenario, etc.) can be found in Table S1. Strains were grown for genomic DNA (gDNA) extraction as follows. Strains were inoculated from freezer stocks into lysogeny broth (LB) medium and incubated for 16 to 18 h at 37°C with shaking. Then, 500 μL of each culture was pelleted via centrifugation for subsequent gDNA extraction.

### Genomic DNA extraction, library preparation, and next generation sequencing.

Genomic DNA was extracted from 500-μL pellets using Invitrogen’s PureLink genomic DNA kit (Thermo Fisher Scientific, product no. K182002), according to the manufacturer’s instructions. Purified gDNA was quantified and stored at –20C until sequencing library preparation. A total of 250 to 400 ng of gDNA was used for library preparation for each strain. Libraries were prepared using the Illumina DNA prep kit (catalog [cat.] no. 20018705), following the manufacturer’s protocol. Libraries were sequenced on an Illumina MiSeq instrument using paired-end reads, to a depth of at least 30× coverage using the MiSeq V2 and V3 reagent kits (Illumina, cat. no. MS-102-2002 and MS-102-3001). Raw reads and assemblies are available under BioProject PRJNA819016.

### Bioinformatic analysis.

The resultant FASTQ files were downloaded from the instrument, and the quality of the sequencing was evaluated using FastQC v. 0.11.9 with default options. We assessed the read files for quality by looking at the number of sequences with over 85% Q30 base pair reads and overall passing all checks for base quality, GC content, N content, length distribution, and overrepresented sequences (90). FASTQ files were analyzed for sequence contamination to ensure that the sequencing information was for one isolate using ConFindr v. 0.7.0 (91). Sequences were assembled using SPAdes v. 1.13, with the options–isolate and paired reads (56). All contigs shorter than 1,000 bp were removed. CheckM v. 1.2.2 was run with the marker set for Escherichia containing 1,628 marker genes across 307 sets using the option taxonomy_wf species to compute assembly statistics on the curated assemblies. Statistics included completeness and percent contamination, and individual statistics are found in Table S1 and summarized in Table 1. FASTG files were input into Bandage v. 0.8.1 to be visualized. Bandage graphs are available in Files S1 to S4.

A phylogenetic tree was constructed using Parsnp v. 1.15 with default options (58). A maximum likelihood tree on the core genome of these isolates was constructed and rooted to the reference genome for cystitis isolate UTI89 (CP000243.1). The tree was visualized using iTOL and annotated according to the respective phylogroup as determined using ClermonTyping v. 26.03 and the clinical scenario of the associated source patient (57, 59, 92).

A list of virulence factors was curated that is frequently associated with uropathogenic and diarrheagenic E. coli strains. We used the VirulenceFinder database as a basis for the diarrheagenic virulence factors and supplemented it with genes described in the literature for other uropathogenic-associated factors (5, 25, 40, 51). Virulence factors in the assembled genomes were identified using BLAST v. 2.12.0+, focusing on virulence factors most frequently associated with pathogenic E. coli. A threshold of 70% identity and 85% alignment length was used to call for the presence of a specific virulence factor gene (62). A complete list of sequences used is found in Supplementary Files. Antibiotic resistance genes were identified using ResFinder (v. 2021-06-30 of the software and v. 2021-03-09 of the database), searching for acquired antimicrobial resistance genes using reference 74. Prophage elements were identified using the web server PHASTER, using the database available in December 2020 (71). PHASTER scores prophage elements as “complete,” “questionable,” or “incomplete” based on the presence of structural genes and homology to prophage sequences in the database. All prophage elements smaller than 10 kb were removed to account for other potentially integrating elements, and any overlapping prophage regions were combined and assigned the highest level of completion of the overlapping elements. Plasmid sequences were extracted using plasmidSPAdes (v. 1.13) (93). Extracted plasmid sequences were also input into ResFinder (v. 2021-6-30) to identify antibiotic resistance genes within the extracted plasmid sequences. A custom BLAST database was created using the plasmid sequences, and the virulence factors were aligned against this database to identify the presence of the virulence factor within a plasmid sequence using the same threshold as that described above, 70% identity and 85% length (62). Mobile elements such as transposons, insertion sequences, and integrative and conjugative elements were identified using MobileElementFinder v. 1.0.0 (29).

Once all elements were identified within the assemblies, prophage elements, plasmids, and other mobile elements were cross-referenced with the virulence factors and antimicrobial resistance genes to check for overlapping features. Virulence factors and AMR genes were considered associated with the MGEs if they overlapped plasmids or prophages. This was identified through a custom Python script (github.com/gracehm). The virulence factors and AMR genes were considered associated with MGEs identified by MobileElementFinder if they overlapped within 31 kb upstream or downstream of the identified element, suggesting it could be mobilized. This region correlates with the size of the largest composite transposon within *Enterobacteriaceae* in MobileElementFinder (Tn*6108*) (29, 75). We additionally checked edge cases where annotations for MGEs might not be resolved by counting the number of AMR genes and virulence factors within 31 kb of the end of the contig.

### Statistical analysis.

Statistical analyses (see figure legends for details) were carried out using GraphPad Prism v. 9. Graphs were created using GraphPad Prism v. 9 and Microsoft Excel. Nonparametric Mann-Whitney *t* tests were used for all ASB versus UTI comparisons. Kruskal-Wallis with Dunn’s multiple-correction tests were used in phylogroup comparisons. Two-way ANOVA tests were used to compare MGEs and symptomatology together. A Chi-squared test was preformed to test the association of the presence of a virulence gene with the presence of symptoms of the source patient using 1 degree of freedom.

### Data availability.

All raw sequencing reads were deposited in the NCBI Sequence Read Archive (SRA) under BioProject no. PRJNA819016. All assemblies produced from this work are also deposited under BioProject no. PRJNA819016. For a list of BioSample accession numbers for each sample, please refer to Table S1. Extracted plasmid sequences have been deposited in GenBank.
