# Supplementary figures and images for: The Role of Mobile Genetic Elements in Virulence Factor Carriage from Symptomatic and Asymptomatic Cases of Escherichia coli Bacteriuria
Source: Microbiol Spectr. 2023 May 17;11(3):e04710-22. doi: 10.1128/spectrum.04710-22 (PMC10269530; doi:10.1128/spectrum.04710-22)

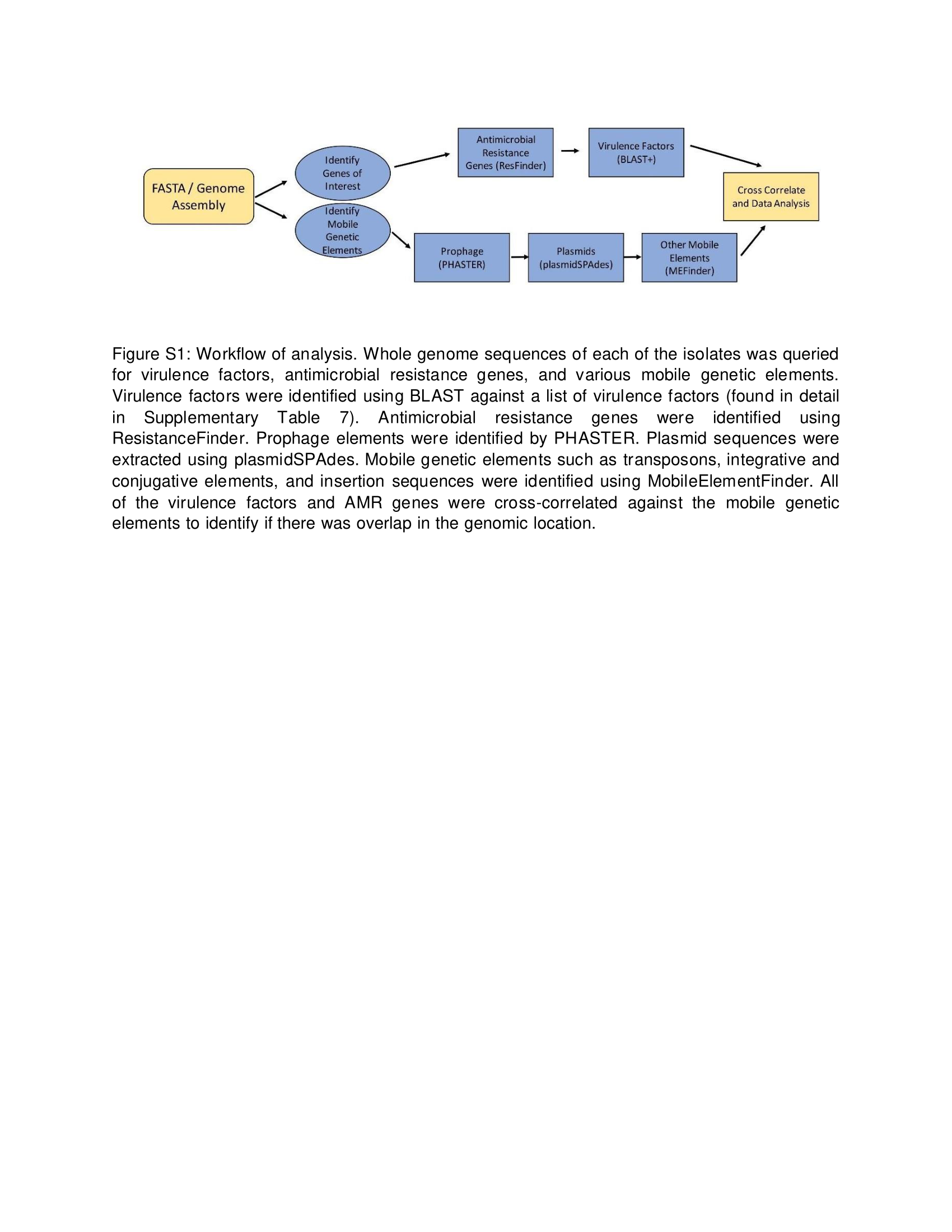

Supplement: Supplemental file 2 — Figures S1 through S5. Download spectrum.04710-22-s0003.tif, TIF file, 1.5 MB [file spectrum.04710-22-s0003.tif]
